# Necessary, Legendary and Detrimental Components of Human Colorectal Organoid Culture Medium: Raising Awareness to Reduce Experimental Bugs

**DOI:** 10.3390/cancers18020337

**Published:** 2026-01-21

**Authors:** Roberto Benelli

**Affiliations:** Struttura Semplice Dipartimentale Oncologia Molecolare e Angiogenesi, IRCCS Azienda Ospedaliera Metropolitana, Largo Rosanna Benzi 10, 16132 Genova, Italy; roberto.benelli@hsanmartino.it

**Keywords:** colon, organoid, culture medium, A83-01, SB202190, nicotinamide, n-acetylcysteine, prostaglandin E2, Primocin

## Abstract

The creation of a specific culture medium for colorectal organoids in 2011 heralded a new era in human primary cultures by enabling the indefinite expansion of normal and pathological epithelial organoids. The original formula has been used ever since, with only minor, lab-specific modifications. The goal of culturing organoids from different tissues has relied on saving and propagating the pluripotent stem cell. The “magic bullet” and all its subsequent derivatives have pursued this goal. Consequently, agonist and antagonist signals are chronically activated in the organoid medium, forcing organoid cells (as well as any other co-cultured cellular model) into constrained signaling pathways. This extremely artificial condition is often overlooked in experimental approaches and may bias the results. Furthermore, some molecules in the organoid medium have unpredictable off-target effects that significantly impact the behavior and maturation of certain cell populations. Nicotinamide, gastrin and PGE2 inhibit immune responses. SB202190, A83-01 and vanadate (from advanced DMEM-F12) modify intracellular signaling. N-AcetylCysteine and Primocin modify the redox response and mitochondrial metabolism, respectively. Thus, the unintentional addition of these molecules to the organoid medium introduces biases under specific experimental settings. While the original organoid medium formula is the gold standard for propagating organoids in vitro, more focused, reliable conditions are necessary for specific organoid-based tests.

## Simple Summary

The invention of a culture medium that can propagate human colorectal organoids indefinitely is a milestone in the development of advanced physiological and pathological models. Although this medium is highly effective, its formula contains molecules that affect cell behavior through direct and off-target effects. Unfortunately, most researchers are unaware of the potential risks these molecules pose to their assays. This short review aims to provide a simple and effective overview of the multiple pathways and biological models that are actively modulated by the organoid medium. It also suggests which components should be eliminated under specific conditions to avoid biased results.

## 1. Introduction

Since the first publication by Sato and Clevers in 2011, which reported a reliable method to establish and propagate human colorectal organoids, these models have been used exponentially. A PubMed search (2 January 2026) using the keyword “human organoids” produced 477 records from 2011 to 2015, 4242 from 2016 to 2020, and 13,595 from 2021 to 2025. According to the slope of the curve of published papers, this trend is far from reaching its plateau. Thus, organoids are expected to become the reference model for most physiological and pathological studies. As we recently reviewed [1], the increasing use of organoids has led to the development of new methods, models and instruments, as well as ethical challenges. While this pioneering phase of organoid research is extremely stimulating and full of new ideas, it increases the need for standardization to ensure reproducibility and reliable clinical transfer of results [2,3]. To this end, the organoid medium should be the first target of standardization [4,5]. Many studies report the original Hans Clevers’ formula as the standard in their methods (Table 1). However, most authors used this formula blindly, without considering how the medium’s constituents could influence the biological processes they were studying. Strong inhibitory effects were empirically detected in some models (e.g., those triggered by nicotinamide in immunology studies [6,7]), but many other modulations were ignored, introducing potential biases in the interpretation of results.

This short review highlights how the constituents of the colon organoid medium can strongly affect and artificially modify cell responses. First, we will recall the original colon organoid medium formula and explain how it constrains organoids within defined biochemical signaling. Second, we will highlight the in-target and off-target effects of all constituents, emphasizing their critical influence on specific experimental settings. This information could serve as a valuable reference for any test involving organoids. This is particularly true in clinic, where organoids could allow a personalized therapeutic approach if enabled to respond to drugs without medium-driven artifacts.

## 2. The First, Long-Term, Human Colorectal Organoid Culture

In 2011, Sato and Clevers described the first in vitro method for the long-term expansion of normal and neoplastic human colon organoids [8]. This method allowed for the culture of pure epithelial cells embedded in 3D matrix domes, maintaining the stem cell niche indefinitely. The basal medium included advanced DMEM-F12 (a medium designed for culturing cells with low FCS supplementation) with B27 and N2 supplements (both originally developed for neural cells), and N-AcetylCysteine (NAC) (Table 1). The key added growth factors were Wnt3a and R-spondin, agonists of the β-catenin pathway for stem cell propagation; Noggin, replacing colon Gremlin 1 and 2, and neutralizing bone morphogenic proteins (BMP)-mediated differentiation; and EGF empowering Erk1-2 signaling for proliferation and survival. Notably, R-spondin was included in the cocktail following the discovery of its binding to LGR5, the marker of colon stem cells, a few months earlier [23].

Although these growth factors could theoretically suffice for culturing colon organoids, they only permitted short-term propagation. A broad screening of compounds was conducted to refine the formula. The addition of survival modulators (nicotinamide, gastrin, prostaglandin E2 and Y27632) increased the plating efficiency of primary cultures and secondary splits. Two essential signaling inhibitors were also identified: the anaplastic lymphoma kinase 5 (ALK5) inhibitor A83-01, which blocks TGFβ receptor signaling, and the p38 inhibitor SB202190. This final mix enabled the long-term, indefinite propagation of colon crypts.

One limitation of this formula is the requirement of high concentrations of growth factors (Wnt3a 100 ng/mL, R-spondin 500–1000 ng/mL, Noggin 100 ng/mL, and EGF 50 ng/mL). Additionally, the poor solubility of recombinant Wnt3a in aqueous solutions can result in reduced or absent biological activity [24]. To date, most labs have abandoned the use of recombinant proteins in favor of the conditioned medium of cells that have been transfected to overexpress Wnt3a, R-spondin, or Noggin (Table 1). This modification introduces in the organoid medium an undefined cocktail of additional factors, spontaneously released by the transduced cells. According to the published protocols, up to 80% of the organoid medium volume comes from 4 to 7-day-old cell culture supernatants made with FCS-containing media. This condition differs greatly from the initial formula, resulting in a loss of standardization and prompting modifications that will be discussed in the following chapters. Since the colon medium recipe formed the basis for developing most organoid media used today, these considerations could be extended to primary cultures of many epithelia.

## 3. Signal Transduction Pathways Involved in Colorectal Organoids Propagation

**Figure 1:**
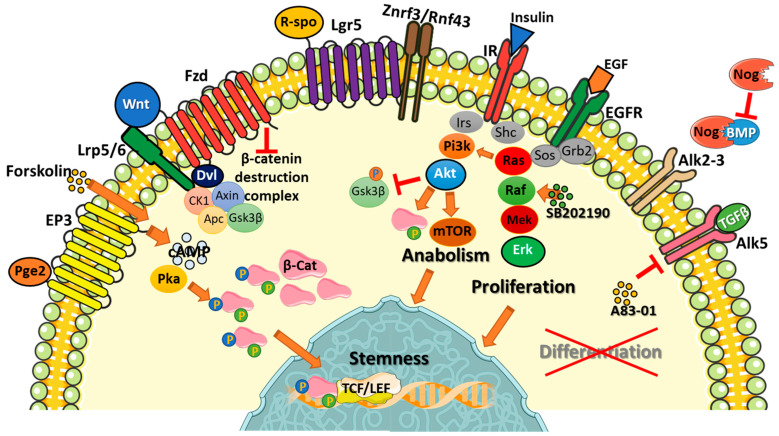
β-Catenin accumulation is triggered by Wnt, which couples the FZD and Lrp5/6 receptors and causes the sequestration of the β-Catenin destruction complex (CK1 + Axin + Apc + Gsk3β). R-spo (R-spondin) binds LGR5 on stem cells and blocks the degradation of the wnt-FZD-Lrp5/6 complex by Znrf3/Rnf43 sequestration, enabling sustained β-Catenin accumulation and signaling. Specific β-Catenin-activating phosphorylations by cAMP/PKA (triggered by PGE2 or Forskolin) and AKT cause protein stabilization and nuclear translocation, thereby enabling TCF/LEF-mediated DNA transcription. The insulin receptor activates both the Pi3K-Akt-mTOR and Ras-Raf-Mek-Erk pathways, the latter empowered by EGFR triggering. The activation of Erk1-2 signaling is also enforced by the off-target RAF-agonist activity of SB202190. Erk1-2 promote cell survival, migration and proliferation and also enforces the β-Catenin transcription of key factors for stemness maintenance. The block of SMAD signaling, necessary to avoid organoid terminal differentiation, is mediated by Nog (Noggin) sequestrating the BMPs released by organoids and A83-01 blocking ALK5/TGFβR1 activity. Brown arrows show positive triggering. Red, upside-down T show inhibitory activity. Pge2 (prostaglandin E2); EP3 (prostaglandin Ep3 receptor); cAMP (cyclic AMP); PKA (protein kinase A); β-Cat (beta-catenin); Lrp5/6 (low-density lipoprotein receptor-related proteins 5 and 6); Wnt (wingless-related integration site); Fzd (frizzled); Dvl (dishevelled); CK1 (casein kinase 1); Apc (adenomatous polyposis coli); Gsk3β (glycogen synthase kinase 3 beta); R-spo (r-spondin); Lgr5 (leucine-rich repeat-containing G-protein-coupled receptor 5); Znrf3/Rnf43 (zinc and ring finger 3/ring finger protein 43); IR (insulin receptor); Irs (insulin receptor substrate); Shc (src homology and collagen-related protein); Pi3K (phosphatidylinositol 3-kinase); Akt (ak strain transforming/protein kinase B); mTOR (mechanistic target of rapamycin); EGFR (epithelial growth factor receptor); Grb2 (growth factor receptor-bound protein 2); Sos (son of sevenless); Ras (rat sarcoma oncogene); Raf (rapidly accelerated fibrosarcoma); Mek (mitogen-activated protein kinase kinase); Erk (extracellular signal-regulated kinase); Alk (anaplastic lymphoma kinase); Nog (noggin); BMP (bone morphogenic protein); TGFβ (transforming growth factor beta).

Before discussing the numerous effects of the components of the colorectal organoid medium, it is important to understand the signaling pathways that affect organoid physiology and viability (Figure 1).

The most difficult endpoint for the colorectal organoid medium is maintaining the LGR5 (leucine-rich repeat-containing G-protein-coupled receptor 5) positive stem cell population. This requires both agonism of positive signals and inhibition of negative signals. The main driver of stem cell persistence and proliferation is β-catenin signaling, which is primarily mediated by the canonical Wnt family members (Wnt 1, 2, 3, 8, and 10). Heavy palmitoylation and glycosylation make Wnts insoluble [25]. This characteristic confers two apparently contradictory effects on these ligands: it confines them to the site of production (allowing the formation of precise gradients along the colorectal crypt axis) and enables them to bind to serum alpha-albumin and lipoproteins, allowing an endocrine-like activity [26,27]. The main effect of canonical Wnt signaling is the destabilization of the β-catenin destruction complex, which allows for β-catenin accumulation and translocation into the nucleus. When CK1 (casein kinase 1), GSK3β (glycogen synthase kinase-3 beta), Axin and APC (adenomatous polyposis coli) assemble the β-catenin destruction complex, CK1 phosphorylates β-catenin at S45 priming successive phosphorylations by GSK3β, directing β-catenin to proteasomal degradation [28]. Wnt binds to and couples FZD (frizzled) and Lrp5/6 (low-density lipoprotein receptor-related protein 5/6) receptors, causing the sequestration and inactivation of the β-catenin destruction complex. β-catenin then accumulates in the cytoplasm and migrates into the nucleus, activating the TCF/LEF (T-cell factor/lymphoid enhancer factor) transcription factor. This enhances the expression of c-Myc (cellular myelocytomatosis oncogene) and Cyclin D, among other genes. Wnt signaling activation is self-limiting. Once β-catenin activates the TCF/LEF transcription factor, E3 ligases (RNF43/ZNRF3) are transcribed early, driving FZD-LRP complex degradation [29]. What differentiates the colorectal stem cells is the binding and activation of LGR5 by R-spondins, causing the sequestration of RNF43/ZNRF3 [30]. R-spondins selectively amplify Wnt signaling in stem cells, activating the ASCL2 (achaete-scute homolog 2) transcription factor, which guarantees the expression of LGR5 and other stem markers [31]. ASCL2 expression is also responsible for regenerating LGR5+ cells in depleted intestinal crypts in mouse models [32], a phenomenon never observed in vitro.

The activation of the TCF-LEF transcription factor by β-catenin does not only rely on the Wnt pathway. The specific phosphorylation of β-catenin at serine residues 552 and 675 by PKA (protein kinase A) promotes its nuclear translocation and TCF-LEF transactivation [33]. Therefore, any increase in cyclic AMP levels could theoretically activate PKA and enhance β-catenin signaling. Akt (Ak strain transforming) is also able to phosphorylate β-catenin on ser552 [34], creating a direct connection between growth factor receptors and Wnt signaling.

β-catenin signaling is necessary, but not sufficient, for maintaining the LGR5+ stem cell. Along the intestinal crypt, an increasing gradient of BMPs (bone morphogenetic proteins) and TGFβ (transforming growth factor beta) drives cell differentiation by SMAD (mothers against decapentaplegic homolog) intracellular signaling. These factors are absent at the bottom of the crypt, where LGR5+ cells are located. BMPs are sequestered by fibroblast-derived Gremlin-1/2 proteins, which act like Noggin as direct BMP trappers [35,36]. Accordingly, SMAD signaling must be strongly limited to prevent the extinction of stem cells in vitro [10].

p38 is a MAPK (mitogen-activated protein kinase) involved in stress and inflammatory responses [37]. Its role in colorectal organoid culture is controversial, but it is generally considered negative, as evidenced by the need for SB202190 (a p38 inhibitor) in the culture medium [8]. A direct consequence of p38 inhibition is the disappearance of Goblet and enteroendocrine cells in cultured organoids [8,38]. However, the presence of these secretory populations did not affect organoid viability, and replacing SB202190 with FGF2 and IGF1 was found to be even more effective in colorectal organoid culture [6]. It appears that the primary beneficial effect of SB202190 is off-target and involves stabilizing EGFR signaling and reducing its degradation [39].

EGFR triggering is the most reliable way to promote organoid proliferation, and EGF is added to almost all organoid media. EGFR directly activates RAS-RAF-MEK-Erk signaling and indirectly activates PI3K-Akt-mTOR signaling [40]. Constitutive Erk1-2 (extracellular signal-regulated kinase 1-2) signaling is necessary for propagating colorectal organoids in vitro by enforcing β-catenin-driven c-Myc and Cyclin D transcription. However, the role of Erk1-2 in the stem cell niche is controversial. Several reports indicate that the absence of Erk1-2 signaling could promote the expansion of intestinal stem cells [41,42], perhaps through compensatory activation of Erk5 [43]. Akt plays a central role in the control of cell metabolism and survival, sensing systemic and local signals to balance cellular anabolism/catabolism [44]. Insulin is always overdosed in organoid medium, pushing cells toward a chronic anabolic phase and triggering Akt activation. Therefore, EGF is not the primary activator of this pathway in vitro. Akt reduces GSK3β activity through direct phosphorylation, which blocks GSK3β-triggered inhibition of CyclinD1 and c-Myc. This allows the cell to cycle [45]. Conversely, a primary downstream effector of Akt, mTOR (mechanistic target of rapamycin), can exert adverse effects by suppressing Wnt/β-catenin signaling and driving LGR5+ stem cells toward exhaustion [46,47].

The NOTCH (neurogenic locus notch homolog protein 1) pathway is another essential actor of intestinal stem cell persistence and absorptive differentiation [48]. Fortunately, organoids can self-sustain this pathway in vitro through cell-to-cell contact, without the need for any external modulators in the culture medium. Jagged peptide, a NOTCH agonist, has been proposed to improve the survival of organoid-derived single LGR5+ cell suspensions [49], but its true relevance has not been definitively proven. Conversely, the NOTCH pathway inhibitor DAPT is used to promote the differentiation of colon organoids toward a secretory phenotype [50].

A frequently neglected signaling pathway is mediated by integrins. Normal colorectal organoids require proper matrix embedding (3D) or coating (2D) to survive and grow [51]. Integrin-mediated signaling is essential for organoid survival and organization, and laminins are critical components of the LGR5+ stem cell niche [52]. Integrins strongly influence growth factor receptor signaling, and vice versa, activating both Erk1-2 and Akt effectors and forming a complementary activation pathway finely modulated by matrix stiffness [53]. The Engelbreth-Holm-Swarm (EHS) mouse sarcoma-derived matrix (Matrigel, Geltrex, Cultrex-BME, etc.) is widely used for organoid cultures. However, some complex polymer-peptide hybrid synthetic matrices show promising results for fully standardized, GMP-friendly applications [54,55].

## 4. Advanced DMEM-F12, B27 and N2

Advanced DMEM-F12, B27 and N2 share several components (see Table 2). An overdose of these components may not necessarily have a positive effect. For example, insulin, transferrin and sodium selenite could be tripled, resulting in high concentrations. Indeed, N2 has gradually disappeared from the formula of the basal medium in many studies, indirectly suggesting that it has an irrelevant or negative influence on organoid growth.

Insulin can have both positive and negative effects on colorectal organoids. Its ability to induce anabolism and protein synthesis through the PI3K-Akt signaling pathway favors cell proliferation [56]. Akt can also reduce GSK3β activity through direct Ser9 phosphorylation. This inhibition does not affect the GSK3β fraction recruited in the β-catenin destruction complex. However, the contemporary activation of Wnt signaling could reinforce overall GSK3β inhibition, increasing β-catenin accumulation [57]. Conversely, a high concentration of insulin can increase mTORC1 activity inhibiting the autophagic turnover of proteins and mitochondria, causing senescence [58,59]. Thus, the benefits and risks of increased insulin concentrations should be carefully evaluated to avoid detrimental effects. Any study involving cell metabolism modulation should also consider this constant excess of insulin.

Ferroptosis is an iron-dependent pathway inducing cell death by mediating non-enzymatic lipid peroxidation [60]. Transferrin receptor 1 is a key mediator of ferroptosis [61], and excess transferrin likely mediates increased intracellular iron levels. However, unless intracellular iron is complexed with ferritin and glutathione (GSH) is abundant, ferroptosis cannot occur. Both advanced DMEM-F12 and B27 contain holo-transferrin (the iron-saturated form) and GSH. However, GSH is a short-lived, spontaneously oxidizing molecule in solution [62] and needs continuous regeneration. The need for constantly high GSH levels to prevent ferroptosis could explain why organoid medium is always supplemented with a high dose of N-acetylcysteine (NAC). NAC is a precursor of reduced glutathione and a direct reactive oxygen species scavenger. Conversely, ferritin is involved in a specific degradation pathway by autophagy (ferritinophagy) mediated by nuclear receptor coactivator 4 (NCOA4) [63,64]. NCOA4 overexpression depletes cellular ferritin content, causing the release of free ferrous ions that can trigger a self-propagating lipid peroxidation. Several factors can induce NCOA4 transcription, including steroid and thyroid hormones, glucocorticoids, cAMP/cGMP and the JNK-JUN pathway [65,66]. These modulators have specific triggers among the organoid medium components (triodo-L-thyronine, progesterone, corticosterone, PGE2, EGF), so an increase in NCOA4 is expected. These observations suggest that transferrin should not be increased in the organoid medium because it could cause negative effects without providing any benefits. Moreover, an equilibrated transferrin content could allow for the reduction or elimination of NAC supplementation, contributing to a simplified organoid medium formula.

Selenium plays an essential role in maintaining redox balance as a coenzyme of glutathione peroxidase and thioredoxin reductase [67]. Cell culture media without FCS are usually deficient in selenium and require a specific supplementation. Despite the fact that seleno-L-methionine has been demonstrated to be less toxic, sodium selenite is used as selenium source [68]. The three-fold increase of sodium selenite in organoid culture medium supplemented with DMEM-F12, B27 and N2 (about 216 nM) is not expected to be toxic, as the toxic threshold for most cellular models is 2.5–10 μM [69,70]. Thus, limited overdosage of this salt should not be detrimental to organoid cultures, even if it is not useful or necessary. Moreover, NAC supplementation could again exert a protective effect [71].

Doubled compounds in the organoid medium are ethanolamine, glutathione, bovine serum albumin, putrescine and progesterone. At this dosage, none of these molecules have a detrimental effect on organoids. However, the presence of doubled progesterone levels should be considered when testing gender-specific effects.

The presence of over-supplemented molecules in the organoid medium is not the only anomaly in biological tests. Some constituents of advanced DMEM-F12 or B27 can strongly modulate cellular responses and affect results in biochemical studies or mixed co-cultures. In particular, ammonium metavanadate and corticosterone are unknown actors in many organoid studies. Vanadate has been identified as a molecule with specific inhibitory activity toward tyrosine phosphatases [72]. Its salts are used to preserve phosphorylated proteins in cell lysates for signal transduction analysis. Several studies have shown that vanadate can increase insulin activity, and many vanadate derivatives have been tested as potential anti-diabetic, anti-parasitic and anti-cancer drugs [73]. While these drugs showed disappointing activity in clinical trials due to their limited tissue distribution, they act as potent triggers in vitro. Vanadate specifically binds to the Fe^2+^ binding pocket of transferrin and forms nonspecific complexes with the His or Asp side chains of several proteins [72,74]. Vanadate inhibits tyrosine phosphatases by binding to the Cys residues in the enzyme active pocket [75]. Consequently, vanadate causes stochastic amplification of signaling pathways involving tyrosine phosphorylation, affecting any experimental system [76]. This mechanism can act as a powerful mechanism of drug resistance against small inhibitors of cell signaling in high-throughput drug screening assays. Although most tests using advanced DMEM-F12 as the basal medium do not consider this bias, a 2010 study showed that treating HeLa cells with EGF or pervanadate could amplify the signaling of 52 and 99 phosphoproteins, respectively, on multiple tyrosine residues [77]. The perturbation of cell signaling mediated by vanadate should be carefully reevaluated in organoid-based assays.

Corticosterone is a component of the B27 supplement, used at a final concentration of 20 ng/mL in the medium, corresponding to 57.8 nM. This dose mirrors corticosterone levels in human plasma (58.4 ± 9.2 nM) [78]. Although corticosterone is a well-known immunosuppressant, exposure to chronically high doses increases the innate immune response, causing cytokine secretion and chronic inflammation [79]. Glucocorticoid neo-synthetic enzymes can be directly induced in the intestinal epithelium by LRH-1 (liver receptor homolog-1), expressed by proliferating cells in the intestinal crypts [80,81]. The activation of this pathway is triggered by immune activation and leads to the final production of corticosterone (in mice) or cortisol (in humans; see [82] for review). Glucocorticoids are also released by CRC cells, participating in the suppression of T-cell activation in this cancer [83]. Exogenous corticosterone in the organoid medium could mask or influence the synthesis of endogenous glucocorticoids in normal and cancerous colorectal organoids. The influence of autocrine glucocorticoid synthesis in human colorectal organoids is a neglected field in immunology.

## 5. Primocin

Primocin is a polyantibiotic mix not included in the original 2011 organoid formula. Although it should be used to prevent contamination during the initial establishment of organoid primary cultures, Primocin is constitutively added to all organoid media. Therefore, the main activity of Primocin is not preventing contamination, but rather having a direct, as yet uncharacterized, positive effect on organoid cultures. In fact, Primocin has been described as a critical additive for culturing and reprogramming both mouse embryonic and human induced pluripotent stem cells [84,85]. Although the composition of Primocin is not fully disclosed, its formula comprises three cell-permeable antibiotics (targeting bacterial DNA gyrase, 30S and 50S ribosomal subunits) and an antifungal targeting ergosterol. Accordingly, we can hypothesize a combination of one fluoroquinolone, one aminoglycoside or tetracycline, one macrolide and either amphotericin or an azole compound. Aminoglycosides, amphenicols, lincosamides, tetracyclines, macrolides, oxazolidinones and streptogramins are known inhibitors of bacterial and human mitochondrial polypeptide synthesis. Thus, the constant presence of these compounds in the culture medium could lower the number of mitochondria and/or their efficiency [86]. Interestingly, high-dose nicotinamide, another organoid medium supplement, also decreases the number of mitochondria, causing their fragmentation and autophagy [87]. This suggests that a reduced number or activity of mitochondria could have positive effects on organoids by lowering reactive oxygen species levels. Any metabolic or metabolomic study should be aware of this constant brake on mitochondria.

Fluoroquinolones are metal cation chelators showing a Fe^3+^ binding ability comparable to deferoxamine [88]. These antibiotics bind to multivalent metal ions by a 1:1 stoichiometry, showing increasing avidity for Mg^2+^ < Mn^2+^ < Zn^2+^ << Fe^3+^ < Al^3+^ [89]. Accordingly, fluoroquinolones could lower the availability of metal coenzymes in vitro and reduce organoid metabolism through direct enzymatic inhibition. This has previously been demonstrated in other cell models for histone and DNA demethylases and collagen prolyl 4-hydroxylases [88]. These biological effects do not produce any plausible advantages in organoid cultures and could cause unpredictable effects during tests. Thus, the long-term exposure of organoids to fluoroquinolones should be avoided.

The undisclosed antifungal compound of Primocin targeting ergosterol could act by direct binding (amphotericin B) or by inhibiting its synthesis (triazoles). Amphotericin B is a powerful molecule directly binding to ergosterol and creating pores in the fungal cell membrane. Unfortunately, it is also highly toxic, as its working concentration in vitro (2.5 μg/mL) is very close to the cell toxicity threshold (5–10 μg/mL) [90]. Amphotericin B also has unpredictable effects on the immune system, activating macrophages and enhancing B, T, NK and Tγδ cell activation in different animal models [91,92,93]. Human colorectal cells (both normal and neoplastic) react to amphotericin B exposure with an active defense mechanism involving the release of amphotericin B-rich exosomes [94]. This effect is also elicited by the standard 2.5 μg/mL dose used in cell culture; thus, Primocin may interfere with studies of exosomes in colorectal organoids. Alternative ergosterol-targeting compounds in Primocin could be triazoles, inhibitors of fungal cytochrome P450 (C-14α-dimethylase). Triazoles bind tightly to the iron ion of this enzyme [95]. Triazoles have been reported to cause 600 severe drug–drug interactions and over 1100 moderate interactions with other drugs, requiring dose modifications in clinical practice [96]. Accordingly, the presence of Primocin could strongly bias any high-throughput assay in drug screening. These observations suggest that Primocin should be eliminated from the organoid medium, especially during experimental assays.

## 6. Wnt3a, R-Spondin and Noggin

Recombinant Wnt3a is frequently inactive due to its low solubility in aqueous solutions [24]. Thus, it is typically supplied to organoids as transfected L-cells (mouse fibroblasts)-derived supernatants. These are prepared by incubating L-cells in an FCS-containing medium for 4–7 days. This additive modifies the original formula by adding a 50% fully metabolized conditioned medium containing FCS, as well as a variety of fibroblast-secreted factors, along with Wnt3a. The situation is even more critical when R-spondin and Noggin are also supplied as HEK cell-derived conditioned media. In this condition, up to 80% of the volume of the original formula is substituted by exhausted cell supernatants. Unfortunately, this broth is effective for organoid culture and has been adopted by leading labs, including that of H. Clevers and many other organoid researchers [12]. As nobody has characterized the real microenvironment created by this dirty formula, it should be noted that unknown factors may contribute to the experimental outcome. To reduce this bias, R-spondin and Noggin could be supplied as recombinant proteins, though this would increase costs. An interesting alternative to Wnt3a is the WNT surrogate [97]. This water-soluble recombinant protein contains the LRP-binding domain of the extracellular Wnt modulator DKK, which is linked to an ankyrin-repeat protein that binds to FZD receptors. Thus, it acts as a bridge between FZD and LRP5/6, just as Wnt proteins do. This clean reagent could be considered a valuable substitute for Wnt3a.

## 7. Nicotinamide

Nicotinamide (Nic) was added at a high dose of 10 mM to the original Sato & Clevers organoid medium formula. Nic was described as a necessary constituent to increase plating efficiency [8]. However, T. Sato eliminated Nic from the formula in successive studies conducted in his lab [13]. Although eliminating nicotinamide did not cause any problems for the organoids, studies inspired by Clevers have continued to use this additive, despite it apparently being unnecessary. On the other hand, Nic exerts several biological activities, modulating SIRT1, PARP and mitochondrial turnover [98,99,100]. The initial inclusion of Nic in the organoid medium formula could be linked to its ability to act as a kinase inhibitor targeting ROCK (Rho-associated coiled-coil kinase) and CK1 [101]. Indeed, Nic mimics the ROCK inhibitor Y27632 (well known by all organoid and stem cell enthusiasts) [102]. Nic inhibits the phosphorylation of myosin light chain, avoiding programmed cell death by anoikis. Nic also acts as a CK1 inhibitor, preventing beta-catenin degradation by the CK1-APC-AXIN-GSK3B destruction complex. In fact, CK1-mediated phosphorylation of the beta-catenin Ser45 residue is mandatory for GSK3B activity and proteasomal degradation [28]. Conversely, the persistence of Nic in culture media has demonstrated growth-inhibiting and pro-differentiating effects on hESC [101].

Although short-term Nic-induced changes could be beneficial for organoid cultures, Nic has been reported to exert opposite effects on immune cells. Nic inhibits T lymphocytes and CART cell cytotoxicity [7,17] and B-lymphocytes activation [103] and reduces dendritic cell super-activation in autoimmune models [104]. However, it can favor the expansion of hematopoietic stem cells [105]. These findings strongly suggest performing Nic-free experiments, particularly in immunological settings.

## 8. N-Acetyl Cysteine

N-acetyl cysteine (NAC) is added to almost all basal organoid medium formulas. However, no study has identified its effects on organoids or justified the selected working concentration. The 1–1.25 mM dose of NAC that is permanently added to the organoid medium exceeds the maximum dose used in short-term treatment for acute acetaminophen intoxication (150 mg/kg ≈ 0.91 mM, 1 h). In a mouse model of ulcerative colitis, long-term administration of a 150 mg/Kg dose of NAC caused irreversible oxidative and inflammatory damage to the colon, liver and kidneys [106]. This effect was mainly mediated by an increase in malondialdehyde (MDA) and an imbalance in TNFα and IL10. A single dose at the same concentration lowered MDA levels in mice treated with organophosphate compounds [107], while a dose of 600 mg twice daily (approximately 0.157 mM) is sufficient to lower MDA in humans [108,109]. In vitro, NAC increases the growth and survival of single circulating tumor cells, but this effect is mediated by an optimized concentration of 0.3 mM [110]. According to these observations, NAC could be overdosed in standard organoid medium. Indeed NAC elimination showed positive effects on prostate organoids [111]. NAC also contrasts with platinum-based chemotherapy [112]. In fact, when NAC was used in drug screenings that included oxaliplatin, no correlation was found between organoid sensitivity and the matched patient response [113,114]). The opposite was observed in the absence of NAC [115]. Thus, NAC should be excluded from any test involving ROS production or evaluating pro-oxidizing drugs.

## 9. A83-01

A83-01 is an inhibitor of ALK5/TGFβR1 and ALK4, ALK7 activin receptors [116]. This small molecule is used in human colorectal organoid cultures at a 0.5 µM concentration to switch off TGFβ receptor signaling. A83-01 blocks Smad2/3 activation, avoiding the terminal differentiation of stem cells. According to Vogt et al., A83-01 is not specific and targets VEGFR and RIPK2 with the same affinity as ALK5 [117]. A83-01 also inhibits MINK1, p38α MAPK and FGFR1 by more than 50% or 30% at a 1 or 0.1 µM concentration, respectively. Because VEGFR inhibition could strongly affect any angiogenesis-related study, A83-01 should be omitted from any multicellular model using endothelial cells. RIPK2 is a master regulator of innate immunity and inflammation and is also involved in T helper activation [118] and rectal cancer progression [119]. Thus, its inhibition should be considered in inflammatory bowel disease models and immune studies. MINK1 is a crossroads for several signal transduction pathways regulating cell cycle, apoptosis, and cell migration [120]. The inhibition of FGFR1 should be considered as an active variable in tests involving organoids alone or in mixed cultures with fibroblasts and endothelial cells. A83-01 has also been shown to promote the growth of mouse hematopoietic stem and progenitor cells in vitro by blocking mast cell differentiation [121]. Although A83-01 is necessary for long-term organoid culture expansion, it can be omitted from short-term assays to eliminate off-target effects.

## 10. SB202190

SB202190 is a p38 inhibitor [122], and its addition to the organoid medium was based on empirical screening of several compounds [8]. P38 inhibition forces colorectal stem cell differentiation toward enterocytes, while secretory Goblet and enteroendocrine cells are no longer represented [8,38]. The absence of secretory populations does not enhance organoid viability. Thus, SB202190 compromises the physiologic development of a multi-population organoid, inducing an undesirable effect.

Despite being a p38 inhibitor, the fundamental activity of SB202190 on colorectal organoids is Erk1-2 signaling amplification [13]. Initially, SB202190-mediated Erk1-2 signaling agonism was attributed to EGFR stabilization and reduced turnover [13]. However, SB202190 can also mimic the BRAF inhibitor Dabrafenib. This results in a well-known paradox: Erk1-2 agonism in BRAF wild-type cells and Erk1-2 inhibition in BRAF-mutated cells [123]. Therefore, SB202190 should not be used with BRAF-mutated CRC organoids. The agonist activity does not rely on EGFR, but rather on increased BRAF/CRAF dimerization induced by the drug [124]. SB202190 also inhibits GSK3β, CK1δ, RIPK2 and GAK off-target, at a concentration of 1 µM, which is far below the 3–10 µM concentration used in organoid cultures [125]. Inhibiting GSK3β and CK1δ can lead to β-catenin accumulation and amplified Wnt signaling. RIPK2, as reported for A83-01, modulates immune responses. GAK (Cyclin G-associated kinase) is involved in clathrin-mediated endocytosis and intracellular trafficking [126], also acting as a positive regulator of mitophagy [127]. Low-dose SB202190 also inhibits MLK2/MAP3K10 and MLK3/MAP3K11, direct modulators of the Jun pathway [128]. At a 10 µM concentration, SB202190 induces autophagy and lysosomal biogenesis by activating PPP3/calcineurin signaling [129]. The multiple, complex activities of SB202190 should be considered in any experimental setting involving organoid biochemistry. This compound should be omitted from short-term tests and from studies of secretory cells.

## 11. EGF, Gastrin, PGE2, Y27632

EGF is a necessary constituent of the organoid medium for culturing cells with a wild-type EGFR pathway. EGF is used at a concentration of 50 ng/mL. This is a high dose that can counteract the efficacy of Cetuximab in vitro [130]. Therefore, when testing Cetuximab, Panitumumab, or other EGFR-targeting antibodies, the optimal EGF dose in the culture medium should be evaluated in advance to avoid false negative results. Although a high dose of EGF is necessary for long-term expansion of normal mucosa organoids, CRC organoids with a wild-type EGFR pathway perform equally well with a 2 ng/mL EGF concentration (our unpublished results), while organoids carrying RAS/RAF activating mutations do not need EGF supplementation. Accordingly, EGF could be lowered or eliminated when testing CRC organoids.

Gastrin, supplemented as [Leu15]-gastrin-I heptadecapeptide, was initially reported to marginally increase organoid plating efficiency (tested in the absence of Nic, A83-01 and SB202190). Gastrin activates the G protein-coupled, cholecystokinin B receptor, which increases organoid swelling through the production and release of HCl [131], a mechanism of swelling shared with PGE2 and Forskolin. The authors originally included gastrin in the culture medium as it “did not interfere with intestinal differentiation” [8]. However, its usefulness has never been confirmed in any subsequent study. Although gastrin is not required in organoid medium, its presence is not neutral, as it could alter the response of different target cells. The cholecystokinin B receptor is expressed in macrophages and B and T lymphocytes, and its triggering usually determines inhibitory effects [132]. Accordingly, gastrin should not be added to organoid medium in immunology tests.

PGE2 is a well-known product of prostaglandin E synthase 2 (PTGS2/COX2), an enzyme strictly associated with CRC progression [133]. Initially, PGE2 10 µM was added to the medium as a rescue molecule after the enzymatic disaggregation of organoids and not as a stable component of the medium [8]. Subsequent studies by Clevers’ group lowered the PGE2 concentration to 10 nM, including it in the standard organoid medium (see Table 1). PGE2 has several positive effects on the colorectal epithelium: it transactivates EGFR signaling, contrasts apoptosis and enforces β-catenin pathway activation [134,135,136]. Moreover, PGE2 mediates tumor-induced angiogenesis [137], and fibroblast-derived PGE2 acts as a trigger for colorectal cancer initiation [138]. These promoting effects on tumor and stromal cells are counterbalanced by the immunosuppressive effects on almost all components of the immune system [139]. The pleiotropic activity of PGE2 should be considered in any experimental approach, and PGE2 should be eliminated from the organoid medium during tests, unless specific studies on its activity are planned.

Y27632 is an inhibitor of the Rho-associated coiled-coil forming protein serine/threonine kinase (ROCK) family. ROCK1-2 mediate the formation of stress fibers in cultured cells by activating the myosin regulatory light chain [102]. Y27632 exerts anti-apoptotic and reprogramming effects on epidermal stem-like cells and pluripotent stem cells cultured without a matrix carrier or stromal support [140,141]. Y27632 increases plating efficiency after the enzymatic disaggregation of organoids and can be stably added to mouse intestinal epithelial cultures to create 2D models [142,143]. Y27632 activity is not limited to ROCK1-2 inhibition; the screening of several kinases revealed that it affects other enzymes involved in cell motility as well (PRK1, PRK2, and ULK1). Moreover, TOPK and PKCε are partially inhibited by a 0.5 µM dose [128]. TOPK is a serine/threonine protein kinase involved in cell cycle regulation and mitosis and is typically upregulated in many cancers [144]. In CRC, TOPK expression is linked to a negative prognosis in patients with KRAS or BRAF mutations [145]. In CRC cells, EGF stimulation induces the PKCε-dependent phosphorylation of migration and invasion inhibitory protein (MIIP), facilitating metastasis [146]. Thus, Y27632 is expected to exert an inhibitory effect on CRC organoid proliferation and invasion. At the standard concentration of 10 µM, Y27632 also partially inhibits AMPK, RSK1, MNK1 and MSK1 [147]. Accordingly, the addition of Y27632 should be limited to the minimal time needed to rescue cell cultures after enzymatic split and should not be added stably during any experimental approach. Of course, Y27632’s intrinsic ability to prevent apoptosis/anoikis does not allow it to be associated with any drug testing.

## 12. Conclusions

**Figure 2:**
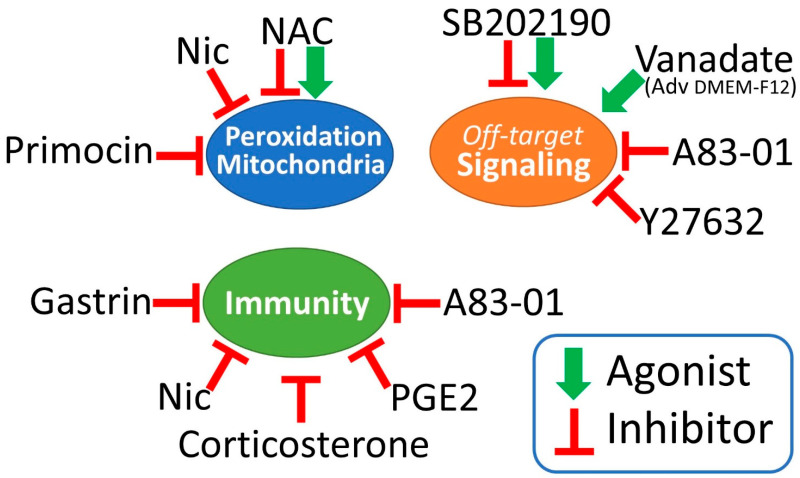
A visual summary of the main experimental biases that could be triggered by different components of the organoid medium. With few exceptions, most additives show inhibitory effects affecting mitochondrial activity and turnover, intracellular signaling and immune cell activation. These molecules should be eliminated or limited, at least in short-term drug testing and immunoassays.

According to the reported information, the colorectal organoid medium is still the gold standard for propagating these unique 3D cultures indefinitely. However, it also creates a highly artificial and incompletely defined microenvironment. Purified growth factors should be preferred to the supernatants from super-expressing cell cultures to prevent the addition of unknown/unwanted paracrine factors. Some components, such as nicotinamide, gastrin, the N2 supplement and Primocin, could be removed from the standard culture medium without issue, thus simplifying the formula and reducing off-target modulations. Other additives, such as NAC, PGE2, A83-01 and SB202190, can significantly impact cell signaling, metabolism, oxidative stress and immunological responses, creating frequent, yet underrated experimental biases (Figure 2 and Supplementary File S1). Unless long-term tests preserving the stem-cell component are necessary, excluding most additives is recommended for short-term, high-throughput assays. This is particularly true when CRC organoids are used for drug screening, as some compounds can create artifactual resistance or sensitization to specific drugs that do not reflect the patient’s situation.

Organoids are now moving beyond basic research into actual clinical applications for personalized cancer therapy [148]. In immunological studies involving monoclonal antibodies, animal models are poor predictors of toxicity, opening the way to organoid testing [149]. Accordingly, the FDA and the NIH are seeking to minimize animal-based models favoring the use of organoids in translational research [150]. Organoid cultures are also changing the approach to studying circulating tumor cells by enabling the culture and expansion of these rare populations in vitro [151]. This approach could enable the identification of residual tumor populations in advance and the prediction of resistance to therapy. Therapy resistance could also be predicted by selectively culturing and testing subclonal tumor populations found in primary organoid cultures [152]. These fields of innovation will create valuable models for personalized medicine, increasing the need for standardization.

This review highlights technical and biological limitations that exist in organoid models based on the artificial nature of the culture medium. Indeed, the various modifications in the components of the organoid medium can cause challenges in standardization that affect interpretation and scientific communication across different fields of precision medicine. Advances in organoid technology have also led to an important and widely used tool for preclinical drug development [153]. But a drug approved solely because of organoid discovery is still emerging. Nonetheless, the promise of cancer drug discovery may occur through a more complete awareness of how the components of organoid medium affect signaling pathways and tumor cell behavior.

## Figures and Tables

**Table 1:** Synopsis of human intestinal organoid culture medium modifications used in different studies.

Medium	Additives	EGF ng/mL	Wnt3a ng/mL; or %CM	R-Spondin ng/mL or %CM	Noggin ng/mL or %CM	A83-01 µM	SB202190 µM	Nic mM	NAC mM	Gast nM	PGE2 µM	Refs.
Advanced DMEM/F12	B27 + N2	50	100	1000	100	0.5	10	10	1	10 **	10 *	[8]
Advanced DMEM/F12	B27noA + N2	50	50% CM	1000 ***	100	**LY2157299** 0.5 μM	10	10	1	48 1 μg/mL	0.01	[9]
Advanced DMEM/F12	B27 + N2	50 **+IGF1** 50 ng/mL	100	500	100	0.5			1			[10]
Advanced DMEM/F12	B27	50	50% CM	10% CM	100	0.5	10		1	10		[11]
Advanced DMEM/F12	B27	50	50% CM or omitted in CRC	20% CM	10% CM	0.5	3	10	1.25	10	0.01	[12]
Advanced DMEM/F12	B27	50	50% CM or omitted in most CRC	10% CM omitted in most CRC	100	0.5	10		1	10		[13]
Advanced DMEM/F12	B27 + N2	50 **+bFGF** 20 ng/mL	omitted (CRC)	omitted (CRC)	omitted (CRC)				1			[14]
Advanced DMEM/F12	B27	50	50% CM or omitted (CRC)	20% CM	10% CM	0.5	10	10	1.25 nM			[15]
Advanced DMEM/F12	B27	50	omitted (CRC)	omitted (CRC)	100	0.5			1	10		[16]
Advanced DMEM/F12	B27	50	50% CM	1000	100	0.5	10		1	10		[6]
Avanced DMEM/F12	B27	50	50% CM or omitted (CRC)	20% CM	10% CM	0.5	10	10	12.5 ****			[17]
Advanced DMEM/F12	B27	50	omitted (CRC)	500	100	0.5	3		1	10	0.01	[18]
Advanced DMEM/F12	B27	50	20% Afamin-Wnt3A serum-free	10% CM	100	0.5	10		1	10		[19]
Avanced DMEM/F12	B27	50 **+FGF10** 100 ng/mL	omitted (CRC)	20% CM	100	0.5	3	10	1.25	10	0.01	[20]
Avanced DMEM/F12	B27	50	omitted (CRC)	omitted (CRC)	100	0.5			1	10		[21]
Avanced DMEM/F12	B27	50 **+bFGF** 10 ng/mL **+FGF10** 10 ng/mL	100	500	100	0.5	5	4		10	0.1	[22]

Legend: **EGF** (epithelial growth factor); **IGF** (insulin-like growth factor); **FGF** (fibroblast growth factor); **Wnt3a** (wingless/int 3a); **A83-01** (TGF-ß type I receptor/ALK5 kinase inhibitor; also inhibits ALK4 and ALK7); **LY2157299** (TGF-ß type I receptor/ALK5 kinase inhibitor; also empowers VEGF and bFGF signaling); **SB202190** (p38 inhibitor); **Nic** (nicotinamide); **NAC** (n-acetylcysteine); **Gast** ([Leu15]-gastrin I); **PGE2** (prostaglandin E2). **CM**: transfected cells conditioned medium. **CRC**: colorectal cancer-derived cultures. * used to increase plating efficiency during the first 48 h of culture; ** originally described to slightly increase plating efficiency, but stably let in the medium formula as it did not cause differentiation; *** home-made RSPO1-His6, purified from HEK293T cells; **** erroneously reported?

**Table 2:** A list of the components of advanced DMEM-F12, B27 and N2.

Reagent	Unique Components	Shared by All	Shared by Two
**Advanced DMEM-F12**	DMEM/F12, Ascorbic acid, Ammonium metavanadate, Cupric sulfate, Manganous chloride, (+Hepes)	Insulin, Holo-Transferrin, Sodium selenite	Ethanolamine, Glutathione, Bovine serum albumin (*AlbuMAX^®^ II*, *lipid-rich*)
**B27 w/wo Vit.A**	Catalase, Superoxide dismutase, Triodo-L-thyronine, L-carnitine, D-galactose, Corticosterone, Linoleic acid, Linolenic acid, Retinol acetate (*not included in the retinol-free formula*), DL-alpha tocopherol, DL-alpha tocopherol acetate, Biotin, Vitamin B12, Zinc sulfate, Selenium, Sodium pyruvate, Lipoic acid, L-Alanine, L-Glutamate, L-Glutamine, L-Proline	Insulin, Holo-Transferrin, Sodium selenite	Ethanolamine, Glutathione-reduced, Bovine serum albumin (*Fraction V IgG free*, *fatty-acid poor*), Putrescine, Progesterone
**N2**	-	Insulin, Transferrin, Sodium selenite	Putrescine, Progesterone

## Data Availability

No new data have been created for this review.

## Supplementary Materials

The following supporting information can be downloaded at: https://www.mdpi.com/article/10.3390/cancers18020337/s1, Supplementary File S1: “Synopsis: effects of the components of the organoid culture medium in different experimental settings”.

## Abbreviations

The following abbreviations are used in this manuscript:

EGF	epithelial growth factor
NAC	n-acetylcysteine
Nic	nicotinamide
PGE2	prostaglandin E2
