# Supplementary material for: Necessary, Legendary and Detrimental Components of Human Colorectal Organoid Culture Medium: Raising Awareness to Reduce Experimental Bugs
Source: Cancers (Basel). 2026 Jan 21;18(2):337. doi: 10.3390/cancers18020337 (PMC12838907; doi:10.3390/cancers18020337)
Supplement: Supplementary file 1 [file cancers-18-00337-s001.zip › cancers-4069780-supplementary.pdf]

Supplementary file S1

Synopsis: effects of the components of the organoid culture medium in different experimental settings.

green = agonism; red = inhibition; ? = hypothesis

Advanced DMEM-F12

- Vanadate strongly affects cell signaling inhibiting tyrosine phosphatases [signal transduction]
- Used along with B27 and N2 brings Insulin to tripled levels [metabolism]

Primocin

- Inhibitor of bacterial and human mitochondrial polypeptide synthesis [metabolism]
- Metal cation chelator, direct enzymatic inhibition [multiple activities]
- ? Amphotericin B [immunity]
- ? Amphotericin B [exosomes]
- ? Triazoles 600 severe drug-drug interactions and over 1100 moderate interactions with other drugs [drug screening]

Wnt3a, R-spondin and Noggin

- ? When added as exhausted cells supernatants [multiple activities]

Nicotinamide

- Inhibits SIRT1 [multiple activities]
- Inhibits PARP [DNA repair]
- Mitochondrial turnover [metabolism]
- Inhibits ROCK [apoptosis/anoikis]
- Inhibits CK1 [signal transduction] [stemness]
- Inhibits T lymphocytes and CART cells cytotoxicity, B-lymphocytes activation and dendritic cells super-activation in autoimmune models [immunity]

N-acetyl cysteine

- Overdosed [redox balance]
- NAC elimination showed positive effects on prostate organoids [proliferation]
- Contrasts with platinum-based chemotherapy [drug testing]

A83-01

- Inhibitor of ALK5/TGFβR1 and ALK4, ALK7 activin receptors [TGFβ/Activin signaling]
- Inhibits VEGFR and RIPK2 with the same affinity as ALK5 [angiogenesis, immunity, IBD]
- Inhibits MINK1 [cell cycle, apoptosis, cell migration]
- Inhibits FGFR1 [fibroblasts/endothelial cells]

SB202190

- p38 inhibitor [differentiation of secretory cells]
- Influences BRAF/CRAF dimerization [Erk1-2 signaling] [BRAF-mutated CRC]
- Inhibits RIPK2 [immunity, IBD]
- Inhibits GAK [intracellular trafficking] [mitophagy].
- Inhibits MLK2/MAP3K10 and MLK3/MAP3K11 [Jun pathway].
- At a 10µM concentration [autophagy, lysosomal biogenesis].

EGF

- At 50 ng/ml counteracts the efficacy of anti EGFR therapeutic antibodies [drug testing]

Gastrin

- Gastrin receptor is expressed by immune cells [immunity]

PGE2

- Transactivates EGFR signaling, contrasts apoptosis, and enforces β-catenin pathway activation [signal transduction].
- Inhibits most components of the immune system [immunity]

Y27632

- Anti-apoptotic [drug testing]
- Inhibits (partially) TOPK [cell cycle],
- Inhibits (partially) PKCε [invasion/metastasis]
